# Dabigatran Associated Leukocytoclastic Vasculitis

**DOI:** 10.1155/2015/616109

**Published:** 2015-12-30

**Authors:** Evangelos Potolidis, Charalampos Mandros, Kalliopi Kotsa, Evdoxia Mitsiou, Dimitris Potolidis, Panagiotis Fanourgiakis

**Affiliations:** ^1^Department of Internal Medicine B, General Hospital of Volos, 38221 Volos, Greece; ^2^Department of Endocrinology and Diabetes, AHEPA University Hospital, 54636 Thessaloniki, Greece; ^3^University of Human Medicine, 68100 Alexandroupolis, Greece

## Abstract

Common side effects of dabigatran are bleeding, bruising, nausea, diarrhea, and abdomen discomfort. Skin reactions were not often noted (<0.1%). We report a case of 70-year-old male who developed dabigatran related skin reaction resistant to usual therapy. Skin biopsy revealed leukocytoclastic vasculitis.

## 1. Introduction

Dabigatran is one of the newer non-Vitamin K antagonist oral anticoagulants (NOAC). Ischemic stroke and bleeding were with these anticoagulants similar to adjusted dose of warfarin (INR 2-3) in patients with nonvalvular atrial fibrillation [1]. Dabigatran is used to treat and prevent deep venous thrombosis and pulmonary embolism and to prevent stroke and systemic embolization. Common adverse reactions are bleeding events, nausea, gastritis-like symptoms, hematuria, and increased ALT. Anaphylaxis and allergic edema are reported in lesser than 0.1% [2]. Cakmak et al. reported a case of leukocytoclastic vasculitis due to dabigatran in a 74-year-old woman with hypertension, coronary artery disease, nonvalvular atrial fibrillation, and ischemic stroke [3]. Our case will be the second published case of leukocytoclastic vasculitis due to dabigatran. The incidence of leukocytoclastic vasculitis is not well known. Studies from Spain showed that leukocytoclastic vasculitis presented in 10–30 persons per million persons per year [4]. Informed consent was obtained from our patient in order to publish his case.

## 2. Case

**Figure 1:**
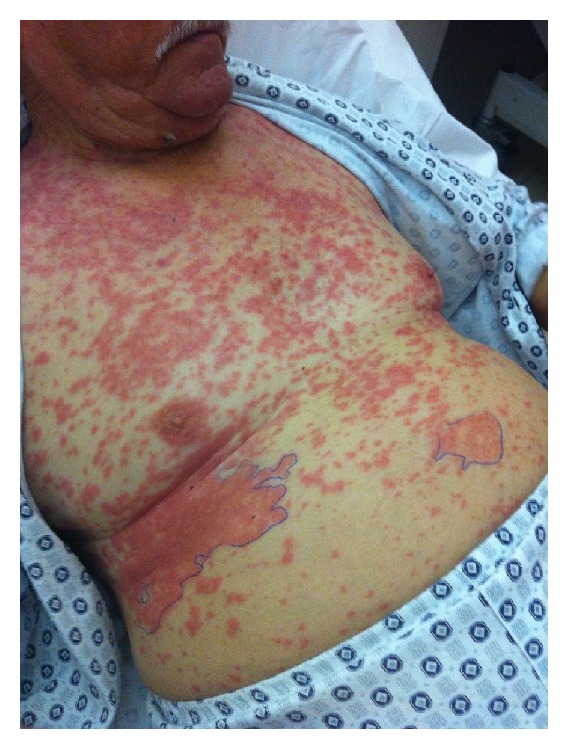


A 70-year-old male was admitted with skin rash on the trunk, back, and limbs (Figure 1). He suffered from nonvalvular atrial fibrillation. The patient did not report any allergies from the past. He took diltiazem 60 mg × 2 in order to lower his heart rate, alprazolam 0,25 mg at bedtime, and dabigatran 150 mg × 2. He was starting taking dabigatran in the previous week. The skin rash developed during the last three days on the trunk and limbs with no response to levocetirizine dihydrochloride which was prescribed by his doctor. He suffered from itching and burning. Conjunctiva, mouth, and genital regions were not involved. On clinical examination, he was found to have arterial pressure 125/85 mmHg, oxygen saturation 95%, heart rate 76 bpm, and normal heart lung auscultation. He did not have fever and his general condition was good. His electrocardiogram showed atrial fibrillation, X-ray was normal, and his hematological and biochemical results were normal. The patient was transferred to the internal medicine department in order to be treated with methylprednisolon 80 mg × 2, dimethindene maleate iv, and H2 antagonists. The eruption was resistant to our therapeutic choices. Immunologic tests were ordered to rule out autoimmune disorders. On the third day of hospitalization skin biopsy was performed, which revealed neutrophilic infiltrates and leukocytoclastic vasculitis. Prednisolon was given 1 mg/kgr as well as colchicine 1 mg/day. On the fifth day of hospitalization the rash was diminished. He was able to leave our clinic two days later. Dabigatran was replaced with enoxaparin 0,6 mL/kgr/12 hours on hospitalization day 1.

## 3. Discussion

Dabigatran is used for prevention and treatment of venous thrombosis and for prevention of ischemic stroke and embolization in nonvalvular atrial fibrillation. Dose reduction is needed (110 mg × 2) in older patients (>75 years) and whenever the clinician estimates increased bleeding risk. Skin reactions due to this drug are very rare. Leukocytoclastic vasculitis is a cutaneous small vessel vasculitis without visceral organ involvement. Drugs such as phenytoin, allopurinol, penicillins, and sulfonamides are able to cause palpable purpura and vasculitis. The same condition could be seen in ANCA positive vasculitis and many infections, like endocarditis, hepatitis virus infections, and HIV. The medications are prone to stimulate the immune response and this will need a time period of one week. Nonblanching lesions and palpable purpura are the main clinical finding, which leads finally to skin biopsy, which reveals neutrophil infiltrates around the vessels, tissue damage, and necrosis. The key point is to exclude ongoing infection or autoimmune disorders with organ involvement. Subsequently first-line treatment is prednisone 0,5–1 mg/kg and second-line agents like colchicine or dapsone can be used in order to relief the patient. Efficacy of colchicine is not well proven [5]. Our patient took for one week dabigatran and developed nonblanching palpable skin lesions which were itching, burning, and resistant to antiallergic treatment options. Dabigatran was discontinued and low molecular heparin replaced. Infection and autoimmune diseases were clinically and immunologically excluded. Skin biopsy revealed cutaneous vasculitis, dabigatran associated, and the patient was effectively treated with prednisolon and colchicine. Clinicians should be informed about this rare adverse effect because of increased use of dabigatran due to its efficacy and good safety profile.

## References

[1] Ruff C. T., Giugliano R. P., Braunwald E. (2014). Comparison of the efficacy and safety of new oral anticoagulants with warfarin in patients with atrial fibrillation: a meta-analysis of randomised trials. *The Lancet*.

[2] Connolly S. J., Ezekowitz M. D., Yusuf S. (2009). Dabigatran versus warfarin in patients with atrial fibrillation. *The New England Journal of Medicine*.

[3] Cakmak M. A., Sahin S., Cinar N., Karsidag S. (2014). Adverse skin reaction caused by dabigatran. *European Review for Medical & Pharmacological Sciences*.

[4] González-Gay M. A., García-Porrúa C. (1999). Systemic vasculitis in adults in Northwestern Spain, 1988–1997. Clinical and epidemiologic aspects. *Medicine*.

[5] Sais G., Vidaller A., Jucgla A., Gallardo F., Peyri J. (1995). Colchicine in the treatment of cutaneous leukocytoclastic vasculitis. Results of a prospective, randomized controlled trial. *Archives of Dermatology*.

